# Antiosteoporotic Effect of Combined Extract of *Morus alba* and *Polygonum odoratum*

**DOI:** 10.1155/2014/579305

**Published:** 2014-11-12

**Authors:** Sudarat Sungkamanee, Jintanaporn Wattanathorn, Supaporn Muchimapura, Wipawee Thukham-mee

**Affiliations:** ^1^Department of Physiology and Graduate School (Neuroscience Program), Faculty of Medicine, Khon Kaen University, Khon Kaen 40000, Thailand; ^2^Integrated Complementary Alternative Medicine Research and Development Center, Khon Kaen University, Khon Kaen 40000, Thailand; ^3^Department of Physiology, Faculty of Medicine, Khon Kaen University, Khon Kaen 40000, Thailand

## Abstract

Due to the limitation of osteoporosis therapy, the alternative therapies from natural sources have been considered. In this study, we aimed to determine the antiosteoporotic effect of the combined extract of *Morus alba* and *Polygonum odoratum* leaves. Ovariectomized rats, weighing 200–220 g, were orally given the combined extract at doses of 5, 150, and 300 mg*·*kg^−1^ BW for 3 months. At the end of study, blood was collected to determine serum osteocalcin, calcium, and alkaline phosphatase level. In addition, tibia bone was isolated to determine bone oxidative stress markers, cortical bone thickness, and density of osteoblast. The combined extract decreased oxidative stress and osteoclast density but increased osteoblast density and cortical thickness. The elevation of serum calcium, alkaline phosphatase, and osteocalcin was also observed. These results suggested the antiosteoporotic effect of the combined extract via the increased growth formation together with the suppression of bone resorption. However, further studies concerning chronic toxicity and the underlying mechanism are required.

## 1. Introduction

Currently, osteoporosis has been regarded as an important health problem. It has been estimated that over 200 million people worldwide suffer from this disease [1]. Osteoporosis, a skeletal disorder characterized by low bone mass and microarchitectural deterioration of bone tissue, increases the risk of fracture. It has been reported that 8 of 20 middle-aged women suffer from one or more osteoporotic fractures during their remaining life time [2]. This condition also produces a substantial burden on annual Medicare costs [3].

In menopausal period, the ovaries stop producing estrogen. Based on the previous findings that estrogen helps to prevent bone loss, the decreased bone mass and density are frequently observed in menopause due to estrogen deficiency [4]. The therapeutic goals for osteoporosis focus on preventing further bone loss and reducing the risk of fracture. Since estrogen deficiency plays the crucial role in the pathophysiology of osteoporosis, hormone replacement therapy (HRT) has been implemented to compensate the deficiency of estrogen. To date, HRT is the gold standard therapy against postmenopausal osteoporosis [5]. However, it has been considered as an unfavorable procedure due to its nonavailability to poor and the rural masses and severe adverse effects. Due to the limitation mentioned earlier, the alternative therapies from natural sources have been considered.

It has been reported that oxidative stress also plays a crucial role in the pathophysiology of osteoporosis. In menopause, the estrogen level declines leading to a loss of protective effect of estrogen against oxidative stress and reactive oxygen species [6] together with the depletion in antioxidant enzymes in bones [7, 8]. In addition, estrogen deprivation also decreases osteoblastic activity but stimulates osteoclastic activity resulting in the development of osteoporosis [9]. Based on the crucial role of oxidative stress in osteoporosis mentioned earlier, the antiosteoporotic effect of herbs possessing antioxidant activity has gained much attention.

Herbs have been long used for treating various ailments worldwide. According to the traditional folklore, most of herbs have been prepared as decoction and polyherbal formulation.* Morus alba* L. (family: Moraceae) or Mohn and* Polygonum odoratum* Lour (family: Asparagaceae) or Pak Paew are widely consumed in the northeast region of Thailand. It has been reported that both of them possess potent antioxidant activity [10, 11] and contain quercetin [10, 12, 13], a substance which provides bone health. Based on the polyherbal concept in traditional folklore, we have previously tested the antioxidant effect of the combined extract of* M. alba* and* P. odoratum* leaves. Our previous results showed that the combined extract provided more potent antioxidant activity than the leaves extract of either* M. alba* or* P. odoratum* (under the patent process). Based on the antioxidant activity, quercetin contents in both plants, and the polyherbal concept in traditional folklore, the bone health benefit of the combined extract of* M. alba* and* P. odoratum* leaves has been considered. Since no supported evidence is available, we aimed to determine the antiosteoporotic effect and its underlying mechanism of the combined extract of* M. alba* and* P. odoratum* leaves in ovariectomized rats, an animal model of menopause.

## 2. Materials and Methods

### 2.1. Plant Preparation

*Morus alba* leave and aerial part* P. odoratum* were collected from Khon Kaen province, Thailand. These plants were cut in small pieces and dried at 60°C and then boiled at 60°C for 25–30 minutes and filtered through nylon cloth. Two fractions of the extracts were lyophilized and prepared as the combined extract at a ratio of 1 : 2 (this ratio provided the highest antioxidant activity). The percent yield of* M. alba *is9.07% and of* P. odoratum* is 8.71%.

### 2.2. Determination of Total Phenolics and Flavonoids Contents

Total phenolic compounds were determined by using Folin-Ciocalteu method [14]. In brief, 0.2 mL (1 mg/mL) of* A. occidentale* leaves extract was mixed with the solution containing 500 *μ*L of Folin-Ciocalteu reagent (10% v/v), 0.8 mL of sodium carbonate (7.5% w/v), and 0.5 mL of distilled water (DW). Then, the mixture was incubated in dark room at room temperature for 45 min. The absorbance was read with an ultraviolet-visible spectrophotometer at 765 nm. DW was used as blank. The sample was prepared in triplicate and mean value was used for the analysis. The total phenolics content (TPC) was expressed as mg of gallic acid equivalent (GAE)/g of dry weight by using the standard calibration line of gallic acid.

Total flavonoid content (TFC) was measured by aluminum chloride colorimetric method [15]. The solution containing 0.5 mL of the plant extract (2 mg/mL), 1.5 mL of 50% alcohol, 0.1 mL of aluminum chloride (10% v/v), 0.1 mL of 1 M potassium acetate, and 2.8 mL of DW was incubated at room temperature for 40 min in dark room. After the incubation, absorbance at 415 nm was recorded by using an ultraviolet-visible spectrophotometer. DW was used as the blank and the sample was prepared in triplicate. Mean value of the triplicate was used for analysis. The total flavonoid content was expressed as mg of quercetin equivalent (QE)/g of dry weight by using the standard calibration line of quercetin.

### 2.3. Experimental Animals and Treatment

Forty-two female Wistar rats, weighing 200–220 g, were obtained from the National Laboratory Animal Center, Salaya, Nakhon Pathom. After the 2-week acclimatization period, they were randomly divided into seven groups (6/group) as follows.

*Group 1*. Control group: rats in this group were orally given vehicle or distilled water (DW) for 3 months. 

*Group 2*. Sham operation plus vehicle treated group: sham operation was performed on all animals in this group and they were orally given vehicle for 3 months. 

*Group 3*. OVX + vehicle (ovariectomized plus vehicle) treated group: rats in this group were ovariectomized and were orally given vehicle for 3 months.

*Group 4*. OVX + genistein (ovariectomized plus genistein) treated group: ovariectomized rats in this group were orally given genistein at dose of 15 mg*·*kg^−1^ BW for 3 months. This group served as positive control.

*Group 5*. OVX + D1 (ovariectomized plus the combined extract at dose of 5 mg*·*kg^−1^ BW): ovariectomized rats in this group were administered the combined extract at dose of 5 mg*·*kg^−1^ BW via oral route for 3 months.

*Group 6*. OVX + D2 (ovariectomized plus the combined extract at dose of 150 mg*·*kg^−1^ BW): in this group, ovariectomized rats were treated as mentioned in Group 5 except that the dose of the combined extract was 150 mg*·*kg^−1^ BW.

*Group 7*. OVX + D3 (ovariectomized plus the combined extract at dose of 300 mg*·*kg^−1^ BW): ovariectomized rats were treated with the combined extract at dose of 300 mg*·*kg^−1^ BW for 3 months.

At the end of study, blood was collected for the determination of serum osteocalcin, calcium, and* alkaline phosphatase levels*. In addition, tibia bone was isolated for the determination of bone oxidative stress markers such as malondialdehyde (MDA) level and the activities of scavenging enzymes including superoxide dismutases (SOD) and* glutathione peroxidase* (GPx). In addition, the cortical bone thickness and density of osteoblast in upper end of tibia bone were also evaluated. All procedures and experimental protocols were approved by the Institutional Animal Ethics Committee of Khon Kaen University (record number AEKKU 16/2013).

### 2.4. Ovariectomy

After 2 weeks of acclimatization, female rats were anesthetized with pentobarbital sodium (50 mg kg^−1^, i.p.), and their ovaries were removed bilaterally. All rats in the sham-operated group were anesthetized, laparotomized, and sutured without removing their ovaries.

### 2.5. Measurement of Serum Calcium, Alkaline Phosphatase, and Osteocalcin

After the anesthesia, blood was collected from abdominal thoracic aorta and centrifuged at 3,000 rpm for 10 minutes. Then, the serum was isolated and kept at −20°C for the determination of serum calcium, alkaline phosphatase, and osteocalcin.

The osteocalcin level was determined by using Rat Gla-Osteocalcin High Sensitive EIA Kit (Takara Bio Inc. Company, Japan). Serum calcium and alkaline phosphatase were determined using standard laboratory method.

### 2.6. Determination of Cortical Bone Thickness and Density of Osteoblast and Osteoclast

The left tibia was removed, cleaned from the adhering muscles, and fixed with PLP fixative (2% paraformaldehyde containing 0.075 M lysine and 0.01 M sodium periodate solution, pH 7.4, stored at 4°C) at 4°C for 24 hr. The tibia bone tissue was dehydrated in a graded series of alcohol and embedded in paraffin wax. The upper end of the tibia was sectioned (5 *μ*m thickness) longitudinally on a rotary microtome and processed for hematoxylin and eosin staining. The thickness of cortical bone and density of osteoblast and osteoclast cells were measured [16].

### 2.7. Determination of Oxidative Stress Markers in Tibia

The left tibia was removed and cleaned from the adhering muscles. The lower end of tibia was used for thedetermination of oxidative stress markers including malondialdehyde (MDA) level and the activities of superoxide dismutases (SOD) and* glutathione peroxidase* (GPx). In brief, tibia was ground as powder and prepared as homogenate by using RIPA buffer solution with protease inhibitors (EDTA) at a ratio of 1 : 10 (w/v). Then it was sonicated at 4°C for 15 minutes to obtain a homogenate. The homogenate was further centrifuged at 1,600 rpm for 10 min [17]. MDA was determined via thiobarbituric acid reaction [18]. Activity of SOD was determined by the method of McCord and Fridovich [19] whereas glutathione peroxidase activity was determined by method of Dundar et al. [20].

### 2.8. Statistical Analysis

All data were expressed as mean ± SEM and analyzed statistically by one-way ANOVA, followed by Tukey's test. The results were considered statistically significant at *P* value <.05.

## 3. Results

### 3.1. The Phenolics and Flavonoids Contents

Total phenolics and flavonoids contents in the combined extract of* M. alba* and* P. odoratum *were evaluated. It was found that the combined extract of* M. alba* and* P. odoratum* leaves contained total phenolics and flavonoids contents at concentrations of 62.42 ± 0.20 mg of gallic acid equivalent (GAE)/g and 32.11 ± 1.21 mg of quercetin equivalent (QE)/g, respectively.

### 3.2. Effect of the Combined Extract of* M. alba* and* P. odoratum* on Serum Calcium, Alkaline Phosphatase, and Osteocalcin Levels

**Figure 1:**
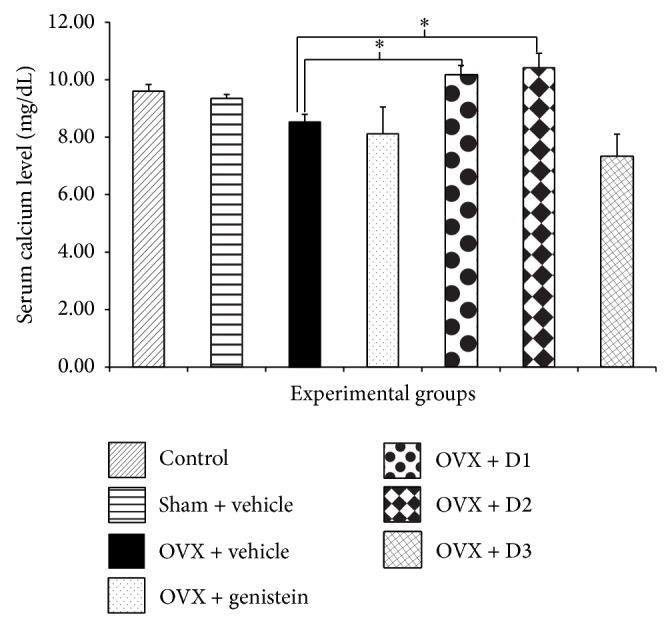
Effect of various doses of the combined extract of* Morus alba* and* Polygonum odoratum* leaves on the levels of serum calcium (*n* = 6/group). ^*^ *P* value <.05, compared to OVX: vehicle treated group. D1: the combined extract at dose of 5 mg*·*kg^−1^ BW, D2: the combined extract at dose of 150 mg*·*kg^−1^ BW, and D3: the combined extract at dose of 300 mg*·*kg^−1^ BW.

**Figure 2:**
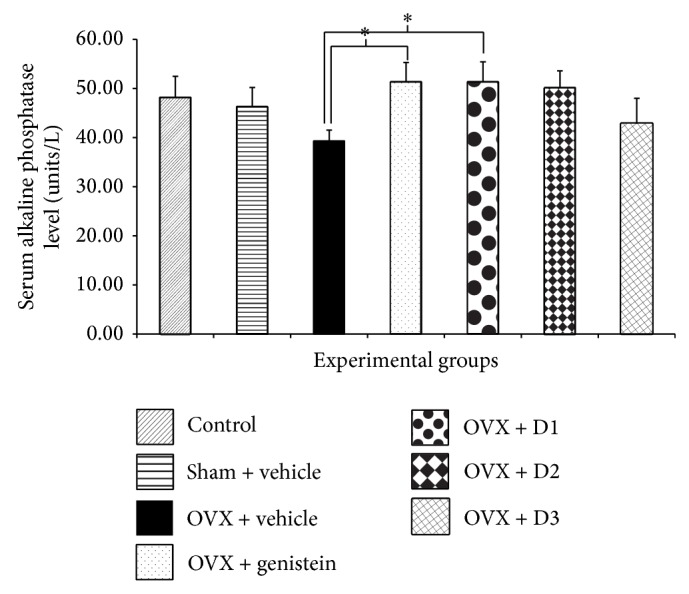
Effect of various doses of the combined extract of* Morus alba* and* Polygonum odoratum* leaves on the level of serum alkaline phosphatase (*n* = 6/group). ^*^ *P* value <.05, compared to OVX: vehicle treated group. D1: the combined extract at dose of 5 mg*·*kg^−1^ BW, D2: the combined extract at dose of 150 mg*·*kg^−1^ BW, and D3: the combined extract at dose of 300 mg*·*kg^−1^ BW.

**Figure 3:**
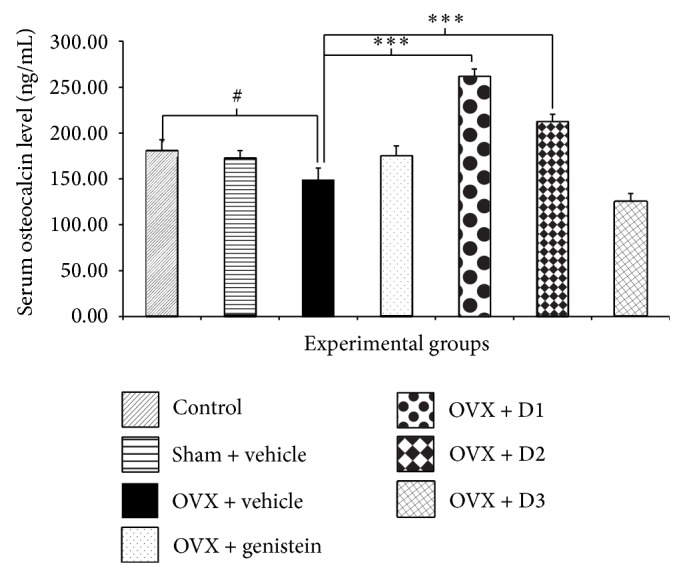
Effect of various doses of the combined extract of* Morus alba* and* Polygonum odoratum* leaves on the levels of serum osteocalcin (*n* = 6/group). ^***^ *P* value < 0.001, compared to OVX: vehicle treated group; ^#^ *P* value <.05, OVX compared to control group. D1: the combined extract at dose of 5 mg*·*kg^−1^ BW, D2: the combined extract at dose of 150 mg*·*kg^−1^ BW, and D3: the combined extract at dose of 300 mg*·*kg^−1^ BW.

Since serum calcium, alkaline phosphatase, and osteocalcin levels could reflect bone metabolic activity to some extent, we also investigated the effect of* M. alba* and* P. odoratum* on serum calcium, alkaline phosphatase, and osteocalcin levels and results were shown in Figures 1, 2, and 3. Serum calcium and alkaline phosphatase were not changed in sham operation group. The serum level of osteocalcin, a bone formation marker secreted from osteoblast, also showed no significant change in sham operation group. Ovariectomized rats decreased all parameters mentioned above. Unfortunately, no significant changes were observed. Ovariectomized rats which received genistein showed only a significant elevation of alkaline phosphatase (*P* value <.05, compared to OVX + vehicle group). Interestingly, ovariectomized rats which received the combined extract of* M. alba* and* P. odoratum* extract at doses of 5 mg*·*kg^−1^ BW significantly enhanced serum osteocalcin, calcium, and alkaline phosphatase levels (*P* value <.001, .05, and .05, resp., compared to OVX + vehicle group). The ovariectomized rats which received the extract at dose of 150 mg*·*kg^−1^ BW failed to show the significant change in serum alkaline phosphatase level. However, the significant elevation of serum osteocalcin and calcium levels was observed (*P* value <.01 and .05, resp., compared to OVX + vehicle group).

### 3.3. Effect of the Combined Extract of* M. alba* and* P. odoratum* on Oxidative Stress Markers

Based on the crucial role of oxidative stress in bone metabolism mentioned earlier [6–9], we also determined the effect of the combined extract of* M. alba* and* P. odoratum* on oxidative stress markers including MDA level and the activities of SOD and GPx enzymes in tibia. The results were shown in Table 1. Sham-operated rats showed no significant changes of all parameters just mentioned when compared to control rats. Ovariectomized rats significantly enhanced MDA level but decreased SOD and GPx activities in tibia (*P* value <.01, .01, and .05, resp., compared to sham-operated group). Ovariectomized rats which received genistein showed only the significant elevation of SOD activities in tibia of ovariectomized rats (*P* value <.05, compared to OVX + vehicle group) but no changes in MDA level and GPX activity were observed. Surprisingly, ovariectomized rats which received the combined extract of* M. alba* and* P. odoratum* at doses of 5, 150, and 300 mg*·*kg^−1^ BW significantly decreased MDA level in tibia (*P* value <.001, .01, and .01, resp., compared to OVX + vehicle group). It was found that ovariectomized rats which received the low dose of extract showed the enhanced GPx activities (*P* value <.01, compared to OVX + vehicle group) whereas rats which received the medium dose of extract showed the significant changes of SOD and GPx activities in tibia (*P* value <.05 and .01, resp., compared to OVX + vehicle group). However, the high dose of extract produced only the significant elevation of SOD enzymes in tibia of ovariectomized rats (*P* value <.001, compared to OVX + vehicle group).

### 3.4. Effect of the Combined Extract of* M. alba* and* P. odoratum* on Bone Histomorphology

**Figure 4:**
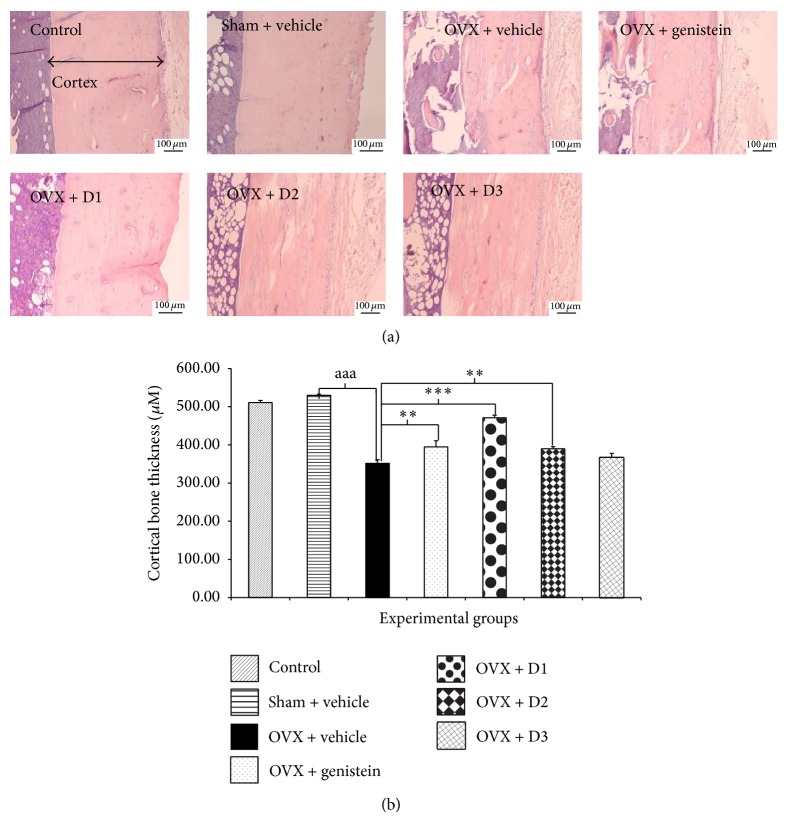
Effect of various doses of the combined extract of* Morus alba* and* Polygonum odoratum* leaves on the cortical thickness of tibia bone. (a) Photomicrograph of longitudinal section of tibia at 5 *μ*m thickness stained with hematoxylin and eosin. (b) Bar graph illustrating the cortical thickness of various treatment groups (*n* = 6/group). ^∗∗,∗∗∗^ *P* value <.01 and .001, respectively, compared to OVX: vehicle treated group. D1: the combined extract at dose of 5 mg*·*kg^−1^ BW, D2: the combined extract at dose of 150 mg*·*kg^−1^ BW, and D3: the combined extract at dose of 300 mg*·*kg^−1^ BW.

**Figure 5:**
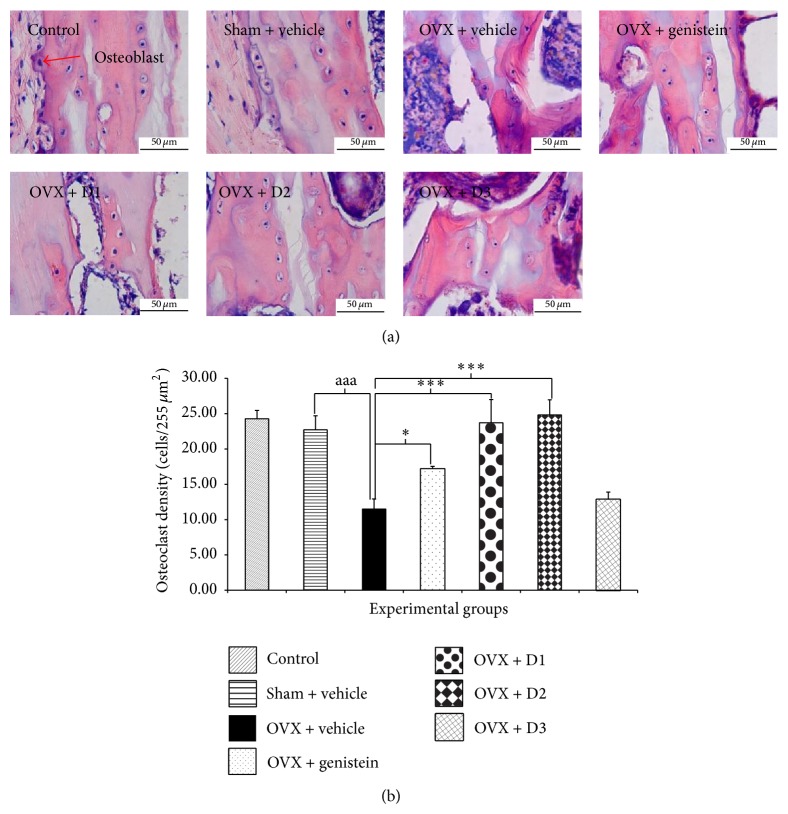
Effect of various doses of the combined extract of* Morus alba* and* Polygonum odoratum* leaves on the density of osteoblast of tibia bone (*n* = 6/group). ^∗,∗∗∗^ *P* value <.05 and .001, respectively, compared to OVX: vehicle treated group; ^aaa^ *P* value <.001, OVX compared to sham operation group. D1: the combined extract at dose of 5 mg*·*kg^−1^ BW, D2: the combined extract at dose of 150 mg*·*kg^−1^ BW, and D3: the combined extract at dose of 300 mg*·*kg^−1^ BW.

The effect of the combined extract of* M. alba* and* P. odoratum* on cortical bone thickness and density of osteoblast cells in tibia was also evaluated and the results were shown in Figures 4 and 5. Rats subjected to sham operation produced no changes of cortical bone thickness and density of osteoblast cells in tibia whereas ovariectomy induced the significant reduction of both parameters (*P* value <.001 all, compared to OVX + vehicle group). The reduction of cortical bone thickness of tibia and density of osteoblast cell induced by ovariectomy was attenuated by genistein (*P* value <.01 and .05, resp., compared to OVX + vehicle group), low dose (*P* value < .001 all, compared to OVX + vehicle group), and medium dose (*P* value <.01 and .001, resp., compared to OVX + vehicle group) treatment. No significant changes were observed in ovariectomized rats which received the treatment of extract at high dose level.

**Figure 6:**
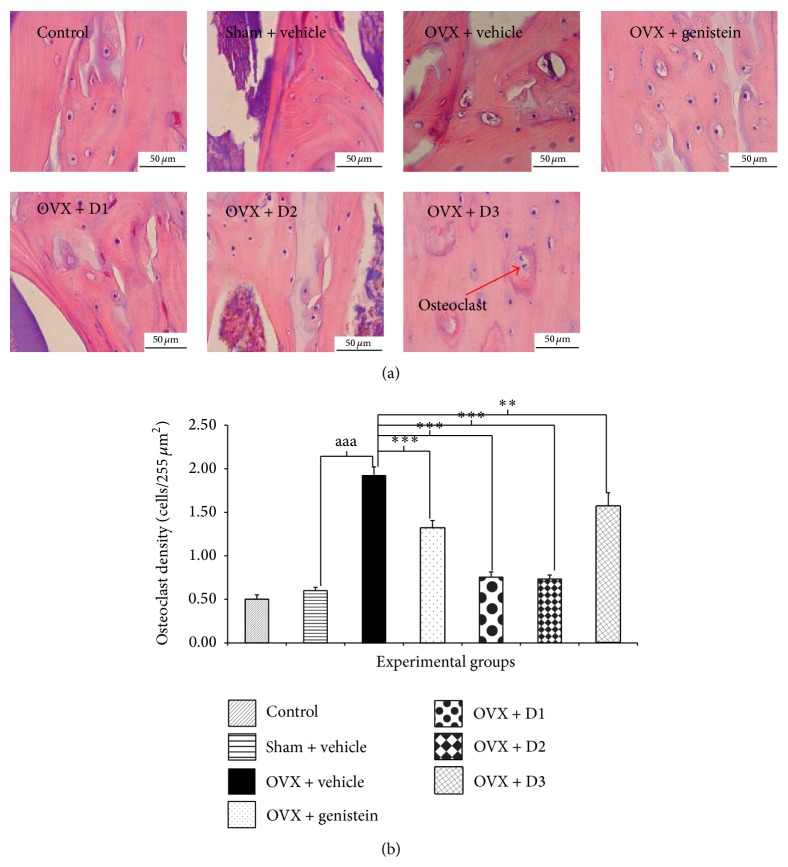
Effect of various doses of the combined extract of* Morus alba* and* Polygonum odoratum* leaves on the density of osteoclast of tibia bone (*n* = 6/group). ^∗∗,∗∗∗^ *P* value <.01 and .001, respectively, compared to OVX: vehicle treated group; ^aaa^ *P* value <.001, OVX compared to sham operation group. D1: the combined extract at dose of 5 mg*·*kg^−1^ BW, D2: the combined extract at dose of 150 mg*·*kg^−1^ BW, and D3: the combined extract at dose of 300 mg*·*kg^−1^ BW.

To make our result more clear, the density of osteoclast was also evaluated.  Figure 6 showed that OVX rats showed the increased osteoclast cell (compared to sham and control). However, the enhanced osteoclast induced by OVX was improved by the low and medium doses of the combined extract of* M. alba* and* P. odoratum*.

## 4. Discussion

The present study clearly reveals that the combined extract of* M. alba* and* P. odoratum* leaves increases osteoblast density, serum osteocalcin, calcium, and alkaline phosphatase levels. The combined extract of* M. alba* and* P. odoratum* leaves also enhances the activities of main scavenger enzymes such as SOD and GPx but decreases MDA level in tibia. In addition, the increased cortical thickness and density of osteoblast in tibia were also observed.

To date, OVX rat model is most commonly used in research on postmenopausal osteoporosis [21]. Ovariectomy disturbs the homeostasis of bone formation and bone resorption giving rise to an excess of bone resorption over bone formation. Our data have shown that ovariectomy could decrease serum calcium, osteocalcin, and alkaline phosphatase together with the increased oxidative stress status. In addition, the reduction of cortical bone thickness and osteoblast induced by ovariectomy was also observed. These changes were not in agreement with the previous studies [21–24]. The possible explanation might be related with difference in stress conditions, physical activity, and hormonal levels which influence calcium absorption and bone regulation such as parathyroid hormone (PTH) [25, 26].

Osteoblasts are specialized fibroblasts that secrete and mineralize the bone matrix. It has been reported that osteoblast, an alkaline phosphatase-rich bone forming cell, plays an important role in osteogenesis via the regulation of matrix deposition [27]. The process of bone growth or maturation of mineral phase is reported to be under the influence of osteocalcin [28]. Therefore, we suggested that the combined extract enhanced osteoblast density and activity and gave rise to the increased cortical bone thickness in OVX rats. The elevation of serum calcium in ovariectomized rats which received the combined extract of* M. alba* and* P. odoratum* leaves observed in this study might be due to the high content of calcium in both plants [29, 30]. This phenomenon might also contribute the role in matrix deposition during osteogenesis.

In this study, ovariectomy also decreases the activities of scavenger enzymes but increased MDA level. This was in agreement with previous study [31]. It has been reported that the elevation of oxidative stress affects both cell viability and the differentiation of osteoblast [32]. Therefore, the antiosteoporotic effect of the combined extract of* M. alba* and* P. odoratum* leaves observed in this study might occur partly via the decreased oxidative stress which in turn increased the survival and differentiation of osteoblast resulting in the increased osteoblast density.

Taken all together, the combined extract of* M. alba* and* P. odoratum* might enhance the activities of scavenger enzymes including SOD and GPX giving rise to the decreased excess oxidative stress indicating by the decreased lipid peroxidation product (MDA). The improved oxidative stress status in the bone in turn increased the survival and differentiation of osteoblast leading to the increased density of osteoblast. Since* M. alba* and* P. odoratum *are rich in calcium, the administration of the combined extract of both plants might also increase serum calcium. The increased density and activity of osteoblast together with the calcium bioavailability induced by the combined extract of* M. alba* and* P. odoratum* leaves then enhanced osteogenesis resulting in the increased cortical bone thickness together with the increased secreted alkaline phosphatase and osteocalcin into the circulation. Based on the antioxidant and antiosteoporotic effect of quercetin, a most common flavonol [33, 34], we suggested that the antiosteoporotic effect of the combined extract of* M. alba* and* P. odoratum* leaves might be associated with the quercetin, the main ingredients in the combined extract.

The dose-dependent manner of bone formation markers was not observed in the ovariectomized rats which received the combined extract. This might occur because no simple linear relationship between the concentration of the combined extract and the observed parameters existed. The tested substance in this study was the combined extract of* M. alba* and* P. odoratum*. Both of them were the crude extract and contained numerous ingredients so the increased concentration could induce the masking effect of inactive ingredient on the antiosteoporotic effect of the active gradient. Moreover the bone remodeling is under the influence of many factors such as bone morphogenetic protein (BMP), hedgehog protein, cell growth factors, cytokine, and hormones [35]. In addition, absorption, distribution, metabolism, and excretion may also exert the influence on the observed parameters. According to this study, the high concentration of extract showed some values less than control and sham because, in control and sham, no estrogen deprivation occurs because all rats in this group still have ovaries. Therefore, the response of the rats was better than the estrogen deprivation rats.

## 5. Conclusion

This study clearly demonstrates that the combined extract of* M. alba* and* P. odoratum* leaves successfully enhances bone formation in animal model of menopause. The possible mechanism might be associated with the decreased oxidative stress status. However, further investigation concerning chronic toxicity and the detailed mechanism are very much essential.

## Figures and Tables

**Table 1:** Effect of various doses of the combined extract of *Morus alba* and *Polygonum odoratum* leaves on the oxidative stress marker in the bone.

Rats groups (*n* = 6)	MDA (nM/mg protein) (mean ± SEM)	SOD (nM/mg protein) (mean ± SEM)	GPx (unit/mg protein) (mean ± SEM)
Control	0.076 ± 0.010	0.161 ± 0.023	1.769 ± 0.186
Sham + vehicle	0.078 ± 0.015	0.130 ± 0.017	1.657 ± 0.272
OVX + vehicle	0.126 ± 0.018^##aa^	0.070 ± 0.015^###aa^	0.869 ± 0.108^#a^
OVX + genistein	0.092 ± 0.008^*^	0.117 ± 0.008^*^	1.162 ± 0.615
OVX + D1	0.060 ± 0.004^***^	0.100 ± 0.007	1.555 ± 0.163^**^
OVX + D2	0.077 ± 0.017^**^	0.111 ± 0.013^*^	1.497 ± 0.169^**^
OVX + D3	0.075 ± 0.015^**^	0.157 ± 0.010^***^	1.143 ± 0.071

(*n* = 6/group) ^∗,∗∗,∗∗∗^ *P* value < 0.05, 0.01, and 0.001, respectively, compared to OVX: vehicle treated group; ^#,##,###^ *P* value < 0.05, 0.01, and 0.001, respectively, compared to control group; ^a,aa^ *P* value < 0.01, 0.001, respectively, compared to sham operation group. D1: the combined extract at dose of 5 mg*·*kg^−1^ BW, D2: the combined extract at dose of 150 mg*·*kg^−1^ BW, and D3: the combined extract at dose of 300 mg*·*kg^−1^ BW.

## Abbreviations

OVX: Ovariectomized
DW: Distilled water
*M. alba*: * Morus alba*
*P. odoratum*: * Polygonum odoratum*
C: Celcius
MDA: Malondialdehyde
SOD: Superoxide dismutase
GPx: Glutathione peroxidase
mg: Milligram
BW: Body weight
D1: Combined extract dose of 5 mg·kg^−1^ BW
D2: Combined extract dose of 150 mg·kg^−1^ BW
D3: Combined extract dose of 300 mg·kg^−1^.
