# Correction to “Antiosteoporotic Effect of Combined Extract of *Morus alba* and *Polygonum odoratum*”

**DOI:** 10.1155/omcl/9864725

**Published:** 2026-03-02

S. Sungkamanee, J. Wattanathorn, S. Muchimapura, and W. Thukham‐mee, “Antiosteoporotic Effect of Combined Extract of *Morus alba* and *Polygonum odoratum*,” *Oxidative Medicine and Cellular Longevity*, 2014, 579305, https://doi.org/10.1155/2014/579305.

In the article, there is an error in Figure 4, in which there are:

• Overlapping regions between the OVX + Vehicle panel and the OVX + genistein panel.
• Overlapping regions between the OVX + D2 panel and the OVX + D3 panel.

The authors have provided a full response and corrected Figure 4 that has been approved by the Chief Editor. The correct Figure 4 is shown below:

Figure 4Effect of various doses of the combined extract of *Morus alba* and *Polygonum odoratum* leaves on the cortical thickness of tibia bone. (a) Photomicrograph of longitudinal section of tibia at 5 μm thickness stained with hematoxylin and eosin. (b) Bar graph illustrating the cortical thickness of various treatment groups (*n* = 6/group).  ^∗∗^, ^∗∗∗^ *p* value < 0.01 and 0.001, respectively, compared to OVX: vehicle treated group. D1: the combined extract at dose of 5 mg⋅kg^−1^ BW, D2: the combined extract at dose of 150 mg⋅kg^−1^ BW, and D3: the combined extract at dose of 300 mg⋅kg^−1^ BW.

**Figure:**
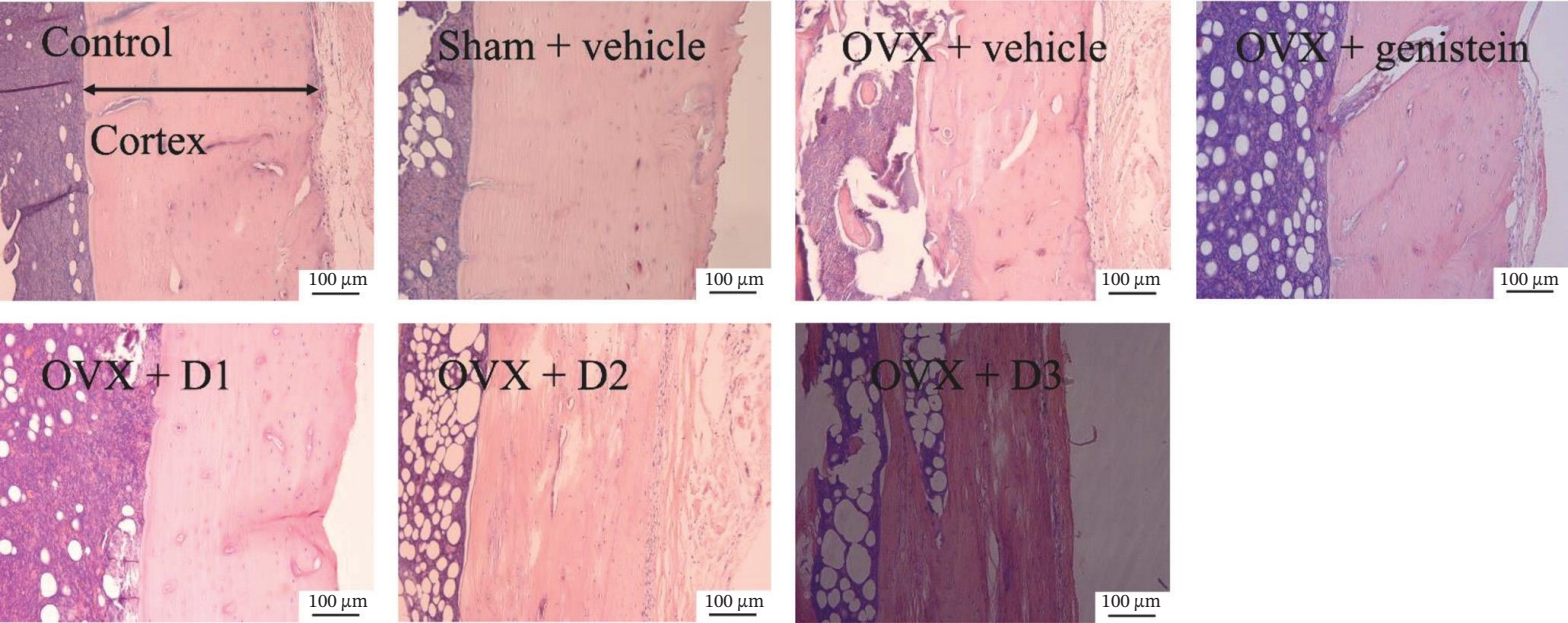
(a)

**Figure:**
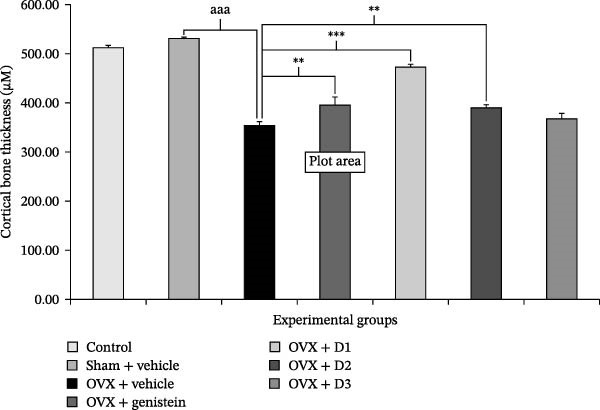
(b)

We apologize for these errors.

